# Gangrene of the Penis

**Published:** 1900-06-16

**Authors:** John M. Longford


### GANGRENE OF THE PENIS.

By John M. Longford, L.R.C.P., &c.

Joseph M., æt. 73, formerly a sailor, was under my care for the past six months with chronic bronchitis and a weak heart. On April 6th he obscurely complained of pain, and then for the first time I became aware of the extraordinary condition of his penis, which I propose to record. The penis presented the appearance of enormous hypertrophy. There was phimosis of evident old standing. The prepuce was very long, and hung over to the right side, completely concealing the glans, with its orifice laterally inclined to the right, as was, in fact, the whole organ. There was much tenderness. The skin on the dorsal and the left lateral aspect of the penis was black, being gangrenous, while remaining portions were in the congestive stage of the same condition. I slit up the prepuce at each side. There was some hæmorrhage, which lessened the congestion and gave some relief to the patient, according to himself. The thickness of the prepuce became then fully evident. It was at least three-quarters of an inch thick, and on left side grooved by the passage of urine, giving the appearance of a divided urethra. The glans was normal. The probable course of events had been as follows: The man had a long prepuce, had had repeated attacks of gonorrhœa, phimosis ensued as a complication, and, being neglected, gave rise to chronic congestion and fibroid thickening of prepuce, and the weak heart was the only factor required to produce gangrene. It must be a rare condition, for it is the only case I have seen here, where, amongst the aged of our workhouse infirmary, senile gangrene is extremely common. The patient died in three days, but a post-mortem examination was not obtainable.

